# Supplementary material for: Content-rich biological network constructed by mining PubMed abstracts
Source: BMC Bioinformatics. 2004 Oct 8;5:147. doi: 10.1186/1471-2105-5-147 (PMC528731; doi:10.1186/1471-2105-5-147)
Supplement: Additional File 2 — The original results of the above study (non-essential files are deleted to keep the file size under the limit set by BMC bioinformatics). [file 1471-2105-5-147-S2.bz2 › chilibotAdditionalFile2/dip20/292ID9485448E1123/html/TRPA_TRPB.html]

 


 **TRPA** and **TRPB** 
  
Found 55 abstracts in PubMed, retrieved 20.  
 

 What does Google say? 
 PDF only 
| .edu only 

---

**Interactive relationship** (e.g. stimulation, inhibition, etc)

**Inhibitory relationship**- These findings suggest that removal of helix 0 of  **TrpA**  decreases  **TrpA**   **TrpB**  association as well as the activity of the  **TrpA**  active site.  Ref: 8662916 J Biol Chem, 1996
**Neutral relationship**- When a 4 bp overlapping leader ORF was placed in front of the successive  **trpB**  and  **trpA**  genes, the  **trpA**  gene was poorly expressed whereas the  **trpB**  gene was overexpressed.  Ref: 12097161 J Biochem Tokyo , 2002
- The third ORF ORF3 consisted of the first 77 nucleotides of the gene encoding the alpha subunit of tryptophan synthase  **trpA** , and overlapped the 3 end of  **trpB**  by 14 nucleotides.  Ref: 8581158 Microbiology, 1996
- Considering the primitive characteristics of C. caldarium,  **trpB**  must have been lost from the plastid genome before  **trpA** .  Ref: 8082179 Curr Genet, 1994

**Non-interactive relationship** (e.g. studied together, co-existance, homology, etc.)

- Complementation analysis in Escherichia coli strains, with defined mutations in tryptophan biosynthesis, and in vitro enzyme activity data, with cloned  **TrpB**  and  **TrpA**  proteins, indicate these mutations result in a  **TrpA**  protein that is unable to utilize indole glycerol 3 phosphate as substrate.  Ref: 12011099 J Biol Chem, 2002
- Poor activity in each reaction was partly due to reduced association of  **TrpA**  with  **TrpB** .  Ref: 8662916 J Biol Chem, 1996
- Three pairs of genes trpG trpD, trpC trpF, and  **trpB**   **trpA**  overlap, and there is intercistronic spacing of 12 6, and 585 bp between the trpE trpG, trpD trpC, and trpF  **trpB**  gene pairs, respectively.  Ref: 1400208 J Bacteriol, 1992
- Unexpectedly,  **trpA**  was not found downstream of  **trpB** .  Ref: 10511537 Genetics, 1999
- One of the most puzzling examples of selection induced mutations involved the simultaneous reversions of two mutations, one in  **trpA**  and the other in  **trpB** , at rates that were several orders of magnitude greater than would have been predicted if the two mutations had occurred as independent events B.  Ref: 8407817 J Bacteriol, 1993
- The alpha and beta reaction activities of independently purified Tk  **TrpA**  and Tk  **TrpB**  were 10 fold lower than the respective activities detected from the Tk TS complex, indicating that during heat treatment, each subunit was necessary for the other to obtain a proper conformation for high enzyme activity.  Ref: 11029579 Eur J Biochem, 2000
- By complementation tests using subcloning of the 9.6 kb NcoI fragments and various E. coli tryptophan auxotrophs, this fragment was found to contain a gene cluster composed of trpE, trpD, trpC,  **trpB**  and  **trpA**  in this order.  Ref: 8956524 Proc Natl Sci Counc Repub China B, 1996
- Differences in patterns of codon usage distinguish the trpI genes of P. syringae and P. putida from P. aeruginosa trpI and from the  **trpB**  and  **trpA**  genes of all three species.  Ref: 8423001 Gene, 1993
- The tryptophan synthases from Escherichia coli and Salmonella typhimurium are tetrameric enzymes, with an elongated  **TrpA** . **TrpB** . **TrpB** . **TrpA**  structure.  Ref: 11209753 Biol Chem, 2000
- The arrangement of genes in the trpCBA cluster is unusual in that trpC is separated by a small open reading frame, trpX, from the potentially translationally coupled  **trpB**  and  **trpA**  genes.  Ref: 10361288 Mol Microbiol, 1999
- In contrast to  **trpA** ,  **trpB**  the gene for the beta subunit of tryptophan synthase was encoded in the cell nucleus.  Ref: 8082179 Curr Genet, 1994
- The trp genes were classified into three linkage groups and they all were closely linked on a short chromosomal region probably in the order  **trpA** ,  **trpB** , trpF trpC, trpD trpE.  Ref: 8586278 FEMS Microbiol Lett, 1995
- Subcloning, complementation of trp strains, nucleotide sequencing of 5.1 kb and 1.95 kb of DNA and subsequent homology comparisons identified the trpC,  **trpB**  and  **trpA**  genes and trpD gene respectively.  Ref: 10361288 Mol Microbiol, 1999
- Transcription of the cloned genes took place from their own promoters, in the following order  **trpB**  > **trpA** .  Ref: 1294446 Genetika, 1992
- Overexpression in Escherichia coli of the AT rich  **trpA**  and  **trpB**  genes from the hyperthermophilic archaeon Pyrococcus furiosus.  Ref: 12435500 FEMS Microbiol Lett, 2002
- Gel filtration chromatography revealed that Tk  **TrpA**  was a monomer alpha, Tk  **TrpB**  was a dimer beta2, and Tk TS complex was a tetramer alpha2 beta2.  Ref: 11029579 Eur J Biochem, 2000
- The  **trpA**  and  **trpB**  genes encoding the tryptophan synthase subunits were cloned from the hyperthermophilic archaeon Pyrococcus furiosus.  Ref: 12435500 FEMS Microbiol Lett, 2002
- The presence of indole, the substrate for the terminal trp pathway enzyme tryptophan synthase, reversed this auxotrophy thereby providing evidence for the existence of an internal promoter independently expressing the tryptophan synthase genes  **trpB**  and  **trpA** .  Ref: 8093062 Biochem Biophys Res Commun, 1994
- To explore the functional role of this region, we analyzed the effects of 19 single and double mutations in  **TrpB**  on the tryptophan synthase TSase and serine deaminase SDase activities of the TrpB2 dimer, either in the presence or in the absence of the  **TrpA**  subunit.  Ref: 11209753 Biol Chem, 2000
- In this study it is shown that, under conditions of intense selection, a strain carrying missense mutations in both  **trpA**  and  **trpB**  reverts to Trp 10 8 times more frequently than would be expected if the two mutations were the result of independent events.  Ref: 2062865 Proc Natl Acad Sci U S A, 1991
- Thus,  **trpB**  and  **trpA**  are regulated positively in three species of fluorescent pseudomonads, P. aeruginosa, P. putida, and P. syringae, but in no other eubacteria so far investigated Crawford, Annu.  Ref: 8423001 Gene, 1993
- Structural studies have identified residues 277 283 of  **TrpB**  as a potentially important region for the allosteric communication between the  **TrpA**  and  **TrpB**  subunits and for the transport of indole between their active sites through a hydrophobic tunnel.  Ref: 11209753 Biol Chem, 2000
- Subcloning and complementation of Escherichia coli trp auxotrophs revealed that as in Brevibacterium lactofermentum, the C. glutamicum trp genes are clustered in an operon in the order trpE, trpD, trpC,  **trpB** ,  **trpA** .  Ref: 7683184 Appl Environ Microbiol, 1993
- Nucleotide sequences of the trpI,  **trpB** , and  **trpA**  genes of Pseudomonas syringae positive control unique to fluorescent pseudomonads.  Ref: 8423001 Gene, 1993
- A 904 bp probe from Pseudomonas aeruginosa was used to identify the  **trpB** ,  **trpA**  and trpI genes of Pseudomonas syringae.  Ref: 8423001 Gene, 1993
- To overexpress broad kinds of GC rich genes in Escherichia coli, we examined how the structures of leader open reading frames leader ORFs affect the expression of GC rich genes, such as polA,  **trpA** , and  **trpB** , from Thermus thermophilus.  Ref: 12097161 J Biochem Tokyo , 2002
